# Echocardiographic Assessment in Patients Recovered from Acute COVID-19 Illness

**DOI:** 10.3390/jcdd10080349

**Published:** 2023-08-15

**Authors:** Luke Stefani, Paula Brown, Monica Gerges, Peter Emerson, Aaisha Ferkh, Kristina Kairaitis, Nicole Gilroy, Mikhail Altman, Liza Thomas

**Affiliations:** 1 Westmead Clinical School, Westmead Hospital, University of Sydney, Westmead 2145, Australia; luke.stefani@health.nsw.gov.au (L.S.);; 2 Cardiology Department, Westmead Hospital, Westmead 2145, Australia; 3 Department of Respiratory and Sleep Medicine, Westmead Hospital, Westmead 2145, Australia; 4 Department of Infectious Diseases, Westmead Hospital, Westmead 2145, Australia; 5 Southwestern Clinical School, University of New South Wales, Kensington 2052, Australia

**Keywords:** COVID-19, echocardiography, GLS, RV free wall strain

## Abstract

Coronavirus (COVID-19) infections have spread rapidly worldwide and posed an immense public health problem. COVID-19 infection can affect the cardiovascular system both acutely and in patients followed up some period after COVID-19 infection. The aim of this study was to evaluate left ventricular (LV) and right ventricular (RV) function by echocardiography in COVID-19 recovered patients (hospitalized and non-hospitalized). Forty-two patients who recovered from COVID-19 but had ongoing symptoms were included in this retrospective observational cross-sectional study. Patients were followed-up at a median time of 112 days from confirmed COVID-19 diagnosis and a comprehensive echocardiogram was performed. COVID-19 patients were age- and sex-matched to healthy controls. Traditional TTE parameters and advanced echocardiographic parameters including two-dimensional LV global longitudinal strain (GLS) and RV free wall strain (FWS) were measured. LV volumes and LV ejection fraction were similar in COVID-19 patients and controls; however, LV GLS was significantly worse in the COVID-19 group (*p* = 0.002). Similarly, RV volumes and traditional RV function parameters were similar, but RV FWS (*p* = 0.009) and RV global strain (*p* = 0.015) were reduced. Alterations in LV and RV strain were observed in both hospitalized and non-hospitalized patients. In the subset of COVID-19 patients without any co-morbidities (*n* = 30), LV GLS remained reduced compared to controls. According to multivariate analysis, COVID-19 infection was the only independent determinant of reduced LV GLS (*p* = 0.012), while COVID-19 infection, diastolic blood pressure, and RV fractional area change were determinants of RV FWS. In this observational study, prior COVID-19 infection demonstrated LV dysfunction in patients with persistent symptoms. Abnormal LV strain was evident in both hospitalized and non-hospitalized patients, suggesting that these changes are independent of the severity of COVID-19 infection at presentation. The use of LV GLS in COVID-19 patients could have potential clinical utility to support the indication for cardiac magnetic resonance imaging in patients with possible COVID-19 related myocarditis. Future longitudinal studies are needed to evaluate its correlation with adverse cardiovascular events.

## 1. Introduction

Coronavirus disease 2019 (COVID-19), from the SARS-CoV-2 virus, spread rapidly worldwide during the pandemic, impacting health and quality of life in individuals as well as at a population level. COVID-19 infection exhibits predominantly respiratory involvement, that varies from mild upper respiratory symptoms to more serious manifestations such as acute respiratory distress syndrome [1]. However, there is evidence that COVID-19 affects multiple organ systems, including the cardiovascular system [2]. Myocardial injury has been documented acutely in hospitalized patients with cardiac abnormalities ranging from asymptomatic elevation of cardiac biomarkers to complications such as cardiac arrest [3,4]. While being a multi-organ disease, understanding the involvement of the cardiovascular system is of importance in determining morbidity and mortality [5,6].

Transthoracic echocardiography (TTE) has shown evidence of regional wall motion abnormalities, bi-ventricular dysfunction, and pericardial effusion during the acute stages of COVID-19 infection [7,8]. Furthermore, patients who sustain myocardial injury (largely determined by elevated troponin levels) during hospitalization have reported continued cardiac abnormalities. The proportion of hospitalized patients with myocardial injury demonstrated by elevated cardiac biomarkers varies from 5% to 38% [9,10], with biomarker-related cardiac injury also having implications for mortality [3]. While it is understood that patients hospitalized due to COVID-19 may have some degree of sustained myocardial injury, it is unknown whether severity of COVID-19 infection at index presentation (i.e., hospitalized vs. non-hospitalized patients), in patients recovered from COVID-19 infection with continued symptoms ≥4 weeks (i.e., “long COVID”), have sustained myocardial injury.

The increased usage of two-dimensional (2D) speckle tracking echocardiography (STE), including left ventricular (LV) global longitudinal strain (GLS) and right ventricular (RV) free wall strain (FWS), may be advantageous in the assessment of myocardial impairment post COVID-19 infection. Thus, the aim of our study is to identify signs of myocardial dysfunction on echocardiogram in patients with “long COVID” and compare them to an age- and sex-matched healthy control group.

## 2. Materials and Methods

The study group comprised of adult patients (≥18 y) who were followed up after an initial presentation with COVID-19 at a tertiary referral center, Westmead Hospital, Sydney, Australia, and included patients admitted to hospital as well as patients who were managed in their homes. Patients were reviewed in the infectious diseases COVID-19 follow-up clinic and referred for echocardiogram if the following requirements were met: (1) patient was previously diagnosed with COVID-19 with a SARS-CoV-2 positive real-time reverse-transcriptase polymerase chain reaction result; (2) patients recovered from acute COVID-19 (≥4 weeks from initial positive result) but continued to exhibit persistent cardiorespiratory symptoms (shortness of breath, fatigue, light-headedness, palpitations, swollen extremities); (3) patients consented to be part of the follow-up study (protocol approved by the area ethics committee (WSLHD HREC no. 2021/ETH12176)). Patients included in this study had been hospitalized due to COVID-19 (*n* = 23) or treated at their home (*n* = 19). Forty patients were diagnosed with COVID-19 during the first wave (2nd March 2020 to 12th of October 2020), with an additional two diagnosed in 2021 (second wave of COVID-19 infection in Australia). Patients did not have any pre-existing lung conditions, nor were they vaccinated, given the timing of COVID-19 infection. The median time for follow-up post confirmed diagnosis was 112 [68.75–178] days. The 42 patients were then age (±3 y) and sex matched with healthy controls identified from the departmental database of our tertiary hospital for comparison.

A comprehensive transthoracic echocardiogram was performed using commercially available ultrasound machines (Vivid E95, General Electric Healthcare, Horton, Norway). All studies were performed by experienced medical professionals or cardiac sonographers. Images were obtained with subjects in left-lateral decubitus position, and acquired from parasternal, apical, and subcostal views using a 3.5-MHz transducer and acquired at high frame rates (>55 fps) [11]. Measurements and recordings were obtained according to the American Society of Echocardiography recommendations [12]. Analysis was performed offline using dedicated software (EchoPac version 203, General Electric-Vingmed, Horton, Norway).

Traditional LV measurements were obtained including biplane LV end-diastolic and end-systolic volumes and indexed to body surface area [12]. Biplane LV ejection fraction was calculated according to Simpson’s method. LV mass was calculated using the Devereaux method and indexed to body surface area [13]. RV end-diastolic and end-systolic volumes and areas were obtained from the apical 4-chamber view. RV ejection fraction and fractional area change (FAC) were calculated. Tricuspid annular plane systolic excursion (TAPSE) was measured using M-mode as the systolic displacement of the lateral tricuspid annulus. Tricuspid lateral annular systolic velocity (R VS’) was measured using tissue Doppler imaging by placing the sample volume on the lateral annular of the tricuspid valve in the apical 4-chamber view. Transmitral pulsed-wave Doppler was performed in the apical 4-chamber view to obtain mitral inflow velocities to assess LV diastolic filling, with the sample volume placed at the mitral leaflet tips [14]. Measurements of mitral inflow included the peak velocities of early (peak E) and late diastolic filling (peak A), and the E/A ratio. Pulsed tissue Doppler imaging was performed placing the sample volume at the septal and lateral mitral annulus in the apical four-chamber view [14,15], obtaining early diastolic (e’) annular velocity. An average of septal and lateral annular e’ velocity was obtained, as recommended [15]. The E/e’ was calculated using average e’ [14]. Left atrial (LA) volume was calculated by modified Simpson’s method from zoomed LA focused apical 4- and 2-chamber views. LA maximal volume (LAVI_max_) was measured at the end of LV systole and indexed to body surface area [12].

LV GLS was measured offline from the 3 apical LV focused views acquired at high frame rate (>60 frames/s). The endocardial border of the left ventricle was traced at end-systole and the region of interest was set to include the LV myocardium. LV systolic strain was measured as the peak negative strain during systole. Strain was calculated as the average of the peak negative global strain during systole from the 4-, 2-, and 3-chamber views. RV FWS was measured offline from the 4-chamber RV-focused view. The endocardial border of the RV was traced, and quality of speckle tracking was confirmed visually from 2D images and strain traces. RV strain was recorded as the peak negative strain during systole of the lateral and septal walls (6 segments). RV FWS was calculated from the 3 free wall segments, and global RV strain was the average of 6 segments. Due to sub-optimal RV-focused images and the retrospective nature of the analysis, seven patients were excluded from RV strain analysis, though all other RV function parameters could be obtained. Although LV GLS, RV global strain, and RV FWS are ‘negative’ values, for simplicity, the absolute values of strain are reported in the results.

Inter-observer variability for LV GLS and RV FWS was performed by two independent operators blinded to measurements in 10 randomly selected patients. Intra-observer variability was performed by the same operator in the same 10 patients at least 4 weeks after the initial measurements. Variability was evaluated by intraclass correlation coefficients (ICC) using a two-way random effects model and a 95% confidence interval; values between 0.75 and 0.9 represented good reliability and values ≥ 0.90 excellent reliability [16].

All analyses were performed using IBM SPSS Statistics version 26 (SPSS, Chicago, IL, USA). The Shapiro–Wilk test was used to analyze the normality of data. Continuous data are expressed as mean ± standard deviation and categorical variables are reported as number (percentages), with Pearson’s Chi-square test used to compare categorical variables between groups. Student’s *t*-test or Mann–Whitney U test was used to compare continuous data when needed.

Relationships between parameters were assessed using Pearson’s or Spearman rank correlation analysis according to data normality. Linear regression analysis was then performed for multivariate analysis. Results were considered significant if *p* < 0.05. Within the COVID-19 group, patients with abnormal LV GLS and RV FWS were compared with those with normal values. Abnormal LV GLS was considered anything worse than 16% and abnormal RV FWS was considered anything worse than 20% [17,18].

## 3. Results

Demographic and clinical data for COVID-19 patients and healthy controls are outlined in Table 1. Thirty COVID-19 patients had no risk factors or prior cardiovascular disease. All patients were in sinus rhythm at the time of the echocardiogram. Those who required oxygen and invasive respiratory support were older than those who did not (59.10 ± 11.38 vs. 42.91 ± 16.64; *p* = 0.007). All patients that required respiratory support (oxygen or otherwise) had two or more cardiac risk factors or a history of AF. All initial demographic and clinical data were similar, apart from increased weight and body mass index (BMI) in the COVID-19 group (*p* = 0.035 and *p* = 0.011, respectively).

Echocardiographic parameters for all COVID-19 and control groups (*n* = 42 in each group) are outlined in Table 2. Inter-ventricular septum thickness was significantly higher in the COVID-19 group compared to controls. LV volumes and LVEF were similar to controls; however, LV GLS was significantly worse in the COVID-19 group. Similarly, RV functional parameters including TAPSE and s’ velocity and FAC were not significantly different, but RV FWS and RV global strain were both lower in COVID-19 patients. Average E/e’ was significantly higher in the COVID-19 group compared to controls; however, only 2 (4.7%) of them had an E/e’ > 14. RV systolic pressure (RVSP) was similar between groups and was within normal limits. As cardiovascular disease or risk factors can independently influence myocardial strain, we performed a sub-group analysis of COVID-19 patients without cardiovascular history or risk factors to controls (*n* = 30) (Table 2). Of all the echocardiographic parameters, LV GLS was significantly lower in the COVID-19 group compared to control group. RV FWS, although reduced in the COVID-19 group, failed to reach significance.

**Figure 1:**
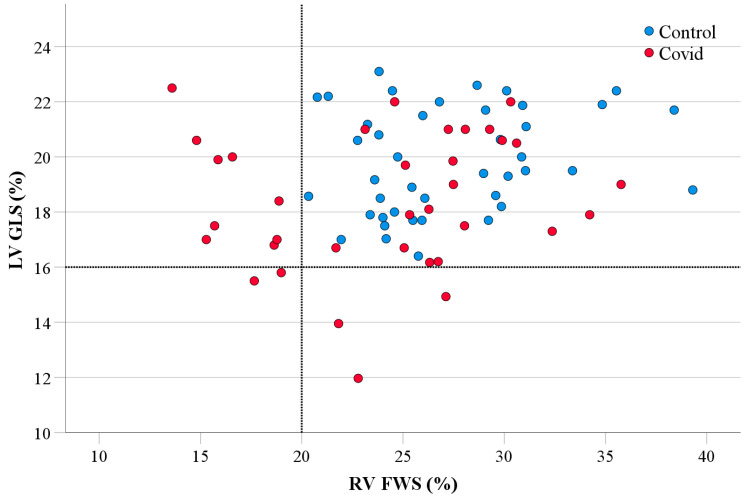
STE assessment of the LV and RV in COVID-19 cohort and healthy controls. Scatter plot demonstrating LV GLS and RV FWS in COVID-19 cohort compared to healthy controls. The horizontal dotted line indicates cut-off for abnormal LV GLS (16%) and vertical dotted line indicates cut-off for abnormal RV FWS (20%). Note: Seven patients had sub-optimal RV focused images, and therefore were excluded from RV strain analysis and not presented on scatter plot. FWS, free wall strain; GLS, global longitudinal strain; LV, left ventricular; RV, right ventricular.

COVID-19 patients were stratified based on previously described cut-offs for ‘normal’ versus ‘abnormal’ LV GLS (<16%) and RV FWS (<20%) (Figure 1). The analysis stratified by LV GLS is presented in Table 3. All COVID-19 patients with cardiovascular history or risk factors, bar one diabetic patient, had normal LV GLS. A total of 20 (55.6%) of the 36 COVID-19 patients with normal LV GLS were admitted to hospital with 3 requiring admission to ICU (8.3%). In contrast, in the abnormal LV GLS group, only 3 (50%) were admitted to hospital and all 3 were admitted to ICU. There was a significantly higher number of patients with abnormal LV GLS requiring invasive respiratory support (*p* = 0.007). Patients with abnormal LV GLS also had significantly higher inter-ventricular septum thickness, LV end diastolic volume, and RV end systolic area.

Subgroup analysis stratified by RV FWS analysis is presented Table 4. Seven patients were excluded from RV strain analysis due to poor image quality or inadequate visualization of the entire RV free wall. A total of 11 (45.8%) of the normal RV FWS group were admitted to hospital with 2 (18.2%) admitted to ICU, while more than half of those with abnormal RV FWS were admitted to hospital with 2 of these patients admitted to ICU. With regard to RV functional parameters, both TAPSE and RVS’ were significantly lower in the abnormal RV FWS group, though FAC was similar between groups (Table 3). Peak E velocity was also significantly lower in this group. The number of patients with abnormal RV FWS who were hospitalized (*n* = 6) was similar to the number who were not (*n* = 5).

Odds ratio analysis is presented in Table 5 for LV and RV strain parameters predicting patients with LVEF < 55% and RV S’ < 9.5 m/s, respectively. In this COVID-19 cohort, patients with an LV GLS < 16% were 8.5 times more likely to have an LVEF < 55%. In addition, COVID-19 patients with an abnormal RV FWS were 9.86 times more likely to have an abnormal RV S’, and patients with abnormal global RV strain were 4 times more likely to have an abnormal RV S’.

For intra-observer variability, the ICC for LV GLS was 0.986 (0.965–0.995) and RV FWS was 0.988 (0.971–0.995). The interobserver variability for LV GLS was 0.962 (0.905–0.985) and RV FWS was 0.948 (0.873–0.979). The results here demonstrate great reproducibility for all strain parameters.

## 4. Discussion

**Figure 2:**
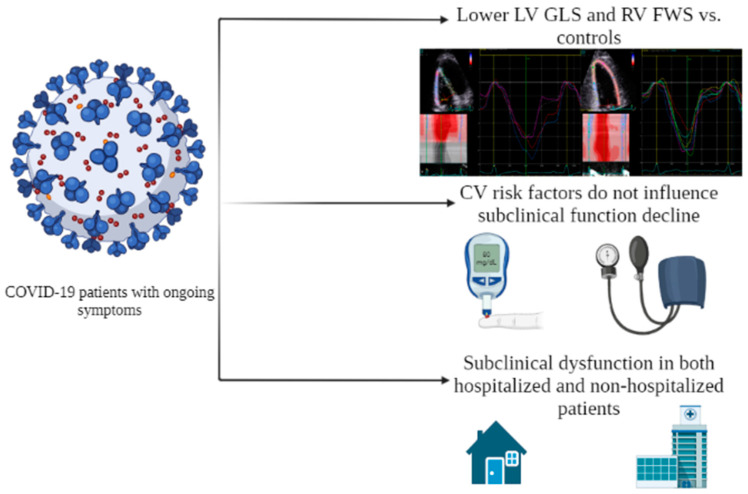
LV and RV function in COVID-19-recovered patients with persistent symptoms. Patients were followed up at the post COVID-19 outpatient clinic and those with persistent cardiorespiratory symptoms were referred for a comprehensive TTE. When compared with age- and sex-matched healthy controls, COVID-19-recovered patients had lower LV GLS and RV FWS. Approximately 55% of patients were admitted to hospital. Patients with CV risk factors did not trend towards abnormal LV GLS and RV FWS groups. CV, cardiovascular risk factors; FWS, free wall strain; GLS, global longitudinal strain; LV, left ventricular; RV, right ventricular; TTE, transthoracic echocardiogram.

In this retrospective observational cross-sectional study, we compared 42 patients with COVID-19 infection during the first COVID-19 wave in Australia, who had ongoing symptoms ≥4 weeks after initial diagnosis, when they presented for review at a COVID-19 follow-up clinic. None of the COVID-19 patients included in this study had received a prior COVID-19 vaccine as this predated the COVID-19 vaccinations. Patients were compared to age- and sex-matched healthy controls for clinical and echocardiographic parameters. The key findings from this study include (Figure 2):

1. The COVID-19 cohort, even without cardiovascular history or risk factors, had lower LV GLS when compared to the healthy controls.
2. Abnormal LV GLS and RV FWS were exhibited almost evenly between hospitalized and non-hospitalized patients.
3. There is a modest correlation between COVID-19 infection and both LV GLS and RV FWS (Tables S1 and S2).

There have been several studies that have assessed the clinical and TTE parameters in patients with acute COVID-19 infection [19,20,21,22,23,24]. The risk factors for developing long COVID-19 are several, including asthma, obesity, age, sex (female), and cardiovascular risk factors (hypertension, diabetes, etc.) [25,26]. Thompson et al. demonstrated in a prospective study that obesity correlated with a 25% higher chance of having prolonged symptoms (>12 weeks) than those who were non-obese [26]. In our cohort, while only 5 (11.9%) patients were obese (BMI > 30 kg/m^2^), the COVID-19 group had a significantly higher weight compared to controls. While BMI was not significantly correlated with LV GLS or RV FWS in our multivariate analysis, obesity and cardiovascular risk factors can promote endothelial dysfunction and inflammation, altering the cardiometabolic limit for exertional symptoms contributing to the persistent symptoms seen in patients with long COVID-19 [27,28]. Additionally, prior cardiovascular history or risk factors within the COVID-19 cohort did not appear to significantly influence the presence of abnormal LV GLS and RV FWS (Table 3 and Table 4). This is particularly apparent for LV GLS in sub-group of COVID-19 patients with risk factors or prior cardiovascular history (Table 2), with LV GLS being the only echocardiographic parameter significantly different between the groups. BMI, although significantly higher in the COVID-19 group, was not an independent predictor of LV GLS within this subgroup. This suggests that the reduction in LV GLS observed in the COVID-19 patients was independent of prior cardiovascular history, risk factors, and BMI.

Cardiac abnormalities have been reported in prospective TTE studies following acute COVID-19 infection. Baykiz et al. followed up hospitalized patients (*n* = 75) post COVID-19 infection (6 ± 1 month), with comparison to an age-, sex-, and comorbidity-matched control group (*n* = 44) [24]. Similar to our results, they observed a lower LV GLS in the COVID-19 group compared to controls (16.7% ± 3.7% vs. 18.3% ± 2.3%; *p* = 0.010); however, they focused specifically on hospitalized patients and did not specify if patients had ongoing symptoms at the time of follow-up. Sudre et al. described data from COVID-19 infected patients (hospitalized and non-hospitalized) with self-reported symptoms at 12 weeks [25]. They demonstrated that patients with prolonged symptoms (>56) days were 37% more likely to be admitted to hospital compared to those who had symptoms for a shorter duration (<10 days) [25]. In contrast, our study includes patients with ongoing symptoms, with only half (*n* = 23 (54.8%)) admitted to hospital during their COVID-19 infection, allowing us to assess severity index of COVID-19 against the relative level of ventricular dysfunction. Interestingly, assessment of LV and RV strain function demonstrated that 26% and 35% of hospitalized patients had an abnormal LV GLS and RV FWS, respectively, as compared to 33% and 28% of non-hospitalized patients. This may suggest that the development of biventricular dysfunction determined by LV GLS and RV FWS is not as influenced by the COVID-19 severity index.

As previously mentioned, myocardial injury has been documented acutely, ranging from asymptomatic elevation of cardiac biomarkers to complications such as cardiac arrest. Given the systemic nature of COVID-19, miRNAs have been proposed as key biomarkers in the prediction of cardiac damage [29]. It has been shown that cardio myocyte and inflammation specific miRNAs that contribute to cardiac fibrosis and hypertrophy are increased in critically ill COVID-19 patients [29,30]. Additionally, increased levels of miRNAs associated with significant endothelial inflammation, fibroblast proliferation, and cardiomyocyte apoptosis (all processes that can lead to ventricular dysfunction and heart failure) have been noted in COVID-19 patients [29]. Hence, it could be postulated that these specific miRNAs could influence the LV and RV strain parameters; however, the current study is limited in answering this as we do not have specific miRNA levels.

This study compares LV and RV function using STE in long COVID-19 patients, including hospitalized and non-hospitalized patients during index COVID-19 infection. Despite the small numbers, we demonstrate that patients exhibit subclinical dysfunction of the LV and RV, regardless of hospitalization status and presence of cardiovascular history or risk factors, that could be surrogate markers of the severity of the index infection. Hence, the inclusion of biventricular strain analysis into a model of care for monitoring and management of patients with long COVID-19 may be pragmatic. Moreover, this may likely identify high-risk individuals with ongoing cardiac involvement, although our cross-sectional study cannot confirm this. While differences in LV and RV strain parameters were apparent, other systolic function parameters and area/volume of the RV and LV were not significantly different compared to healthy controls. Further studies with larger cohorts need to be investigated with longitudinal follow-up to assist clinicians to determine when additional intervention (e.g., cardio protective therapy) is likely to be advantageous for the patient.

### 4.1. Clinical Perspectives

As previously mentioned, potential cardiac complications can arise in vulnerable patients with severe COVID-19 infection. Among these complications is COVID-19 myocarditis, which can present with similar symptoms to COVID-19 infection and develop during or even some time after infection. Although it is infrequent, histopathological findings report varying degrees of cardiac injury in up to 48% of all COVID-19 infected patients [31,32]. This raises the concern for long-term cardiac injury and emphasizes the need for targeted continuous surveillance even after infection [33]. Meindl et al. demonstrates that in patients with cardiac magnetic resonance imaging (CMR)-confirmed acute myocarditis and preserved LV function, there was a significant reduction in LV GLS compared to healthy controls [34]. Extrapolating from this, an abnormal LV GLS in COVID-19 infected patients, during or after infection, could be indicative of potential myocarditis induced by COVID-19. D’Andrea et al. confirmed this in a study demonstrating reduced LV GLS in COVID-19-induced myocarditis when compared to healthy controls [35]. There are a number of guidelines published to aid clinicians’ decisions in recommending CMR for COVID-19-infected patients [32,36,37,38]. The recommendation of CMR in the majority involved prolonged clinical symptoms suggestive of cardiac injury, or previous or high-risk of cardiac injury. The addition of an LV GLS cutoff value could provide potential utility for clinicians to support an indication for CMR.

### 4.2. Limitations

Our study has several limitations and, although carefully performed, included a relatively small number of COVID-19 patients. We enrolled consecutive patients seen in the ‘long COVID-19’ clinic, some of whom had cardiovascular history or risk factors that could independently alter LV and RV strain. However, only one patient with diabetes had an absolute reduction in LV GLS. Additionally, sub-group analysis was performed in COVID-19 patients without any cardiovascular history or risk factors or disease (*n* = 30), and this still demonstrated a significantly lower LV GLS when compared to healthy controls. Unfortunately, we do not have CMR imaging for any of the COVID-19 patients and therefore could not quantify T1/T2 times, extracellular volume (ECV), or myocardial oedema to adequately determine presence of myocarditis.

Patients did not have a TTE prior to or at the time of index presentation with COVID-19 infection, and hence we cannot rule out the possibility of pre-existing abnormal LV and RV strain. Patients received varying therapies, some of which may have an impact on cardiac function; however, numbers were too small to perform meaningful comparisons (Table S3). Our study has a relatively small sample size from a single center and does not encapsulate the entirety of cardiac complications related to long COVID-19. While we assessed both hospitalized and non-hospitalized patients, we only included patients with persistent symptoms. Further evaluation of LV GLS and RV FWS in asymptomatic COVID-19 patients would help determine whether this impairment is only limited to those with persistent symptoms or pre-existing risk factors. As these patients presented in the first wave, cardiac biomarkers including hsTroponin and NT-pro BNP a were only performed in a minority of patients and thus were not included in the analysis. We do not have long term follow-up to determine if the dysfunction observed predisposes individuals to future adverse cardiovascular events. Additionally, there were differences in the therapies received between hospitalized vs. non-hospitalized patients; patient numbers are too small to perform a meaningful subgroup analysis. Our results may not be generalizable to other subtypes of COVID-19 infection, and cardiac involvement may be modified by prior vaccination or other newer therapies such as anti-viral medications.

## 5. Conclusions

This study demonstrated subclinical LV and RV dysfunction in COVID-19 patients with persistent symptoms post-acute phase of COVID-19 when the B.1.1.7 (alpha variant) was predominant in Australia. Hospitalization status at index presentation is not implicated in the observed biventricular dysfunction. Given the limited sample size, findings from this study should only be hypothesis-generating, prompting further examination of dysfunction in the LV and RV in long COVID-19 patients using STE. Further, it is important to determine if such alterations are associated with long-term adverse cardiovascular events. However, evaluation of biventricular strain may assist clinicians in serial follow-up, risk stratification of patients suffering from persistent symptoms after COVID-19 infection, and aid clinicians to perform additional testing including CMR for potential evaluation of COVID-19 myocarditis.

## Figures and Tables

**Table 1:** Demographic and clinical data of healthy controls and COVID-19-infected patients.

	Controls *n* = 42	COVID-19 *n* = 42
Age (y)	46.6 ± 16.4	46.8 ± 16.9
Female	27 (64)	27 (64)
Height (cm)	166.3 ± 8.3	166.3 ± 11.3
Weight (kg)	70 ± 12.5	78.3 ± 21.3 *
BMI (kg/m^2^)	25.3 ± 3.9	28.0 ± 5.4 *
Body surface area (m^2^)	1.78 ± 0.18	1.86 ± 0.29
Systolic blood pressure (mmHg)	122.8 ± 11.2	122.6 ± 15.8
Diastolic blood pressure (mmHg)	74.4 ± 6.9	76.9 ± 9.7
Diabetic	0	2 (4.7)
Hypertensive	0	6 (14.3)
Hypertensive and diabetic	0	4 (9.5)
Ischemic heart disease	0	1 (2.4)
Atrial fibrillation	0	2 (4.7)
Admitted to hospital	0	23 (54.8)
Length in hospital	N/A	7 [2–12]
Admitted to ICU	0	6 (14.3)
Length in ICU	N/A	11.5 [5–49.75]
Required respiratory support (O_2_)	0	10 (23.8)
Endotracheal tube	0	1 (2.4)
Tracheostomy	0	2 (4.8)
Time from infection to echo follow up	N/A	112 [68.75–178]

Mean ± standard deviation and median [interquartile range Q1–Q3] for continuous variables; number (percentage) for categorical variables. * *p* < 0.05. BMI, body mass index; ICU, intensive care unit.

**Table 2:** Echocardiographic data of healthy controls vs. COVID patients.

	Entire Cohort			No Cardiovascular Risk Factors		
Echocardiographic Parameter	Controls *n* = 42	COVID-19 *n* = 42	*p* Value	Controls *n* = 30	COVID-19 *n* = 30	*p* Value
LV systolic function and volumes						
LVEDD (mm)	47.15 ± 4.44	45.45 ± 5.57	0.036	48.27 ± 4.33	45.77 ± 6.25	0.077
LVESD (mm)	30.26 ± 4.82	29.60 ± 6.08	0.315	31.79 ± 4.34	30.27 ± 6.75	0.302
PW (mm)	8.12 ± 1.64	8.76 ± 1.56	0.069	7.89 ± 1.72	8.43 ± 1.59	0.206
IVS (mm)	8.17 ± 1.82	9.17 ± 1.89	0.016	7.90 ± 1.85	8.83 ± 1.90	0.059
LV mass (g/m^2^)	70.90 ± 16.95	73.54 ± 23.09	0.741	70.26 ± 18.60	69.03 ± 24.05	0.826
LVEDV (mL/m^2^)	47.10 ± 10.89	46.70 ± 17.28	0.989	47.89 ± 10.72	47.65 ± 17.73	0.951
LVESV (mL/m^2^)	19.25 ± 5.14	18.91 ± 9.21	0.499	20.05 ± 4.96	19.69 ± 10.09	0.859
LVEF (%)	59.40 ± 5.27	60.19 ± 5.42	0.503	58.43 ± 5.22	59.80 ± 5.72	0.338
LV GLS (%)	19.86 ± 1.90	18.33 ± 2.43	0.004	19.57 ± 1.80	18.10 ± 2.45	0.010
Abnormal LV GLS (<16%)	0	6 (14.3)	0.011	0	5 (16.7)	0.020
LV diastolic function						
Peak E (cm/s)	75.23 ± 15.99	73.14 ± 18.08	0.577	78.44 ± 15.93	77.57 ± 19.26	0.848
Peak A (cm/s)	60.02 ± 15.99	67.55 ± 16.92	0.166	55.73 ± 12.57	61.90 ± 15.92	0.101
Septal e’ (cm/s)	8.75 ± 2.34	7.98 ± 2.88	0.176	9.54 ± 2.10	8.83 ± 2.91	0.286
Lateral e’ (cm/s)	11.88 ± 3.12	10.69 ± 4.68	0.089	12.77 ± 3.00	12.17 ± 4.67	0.557
E/mean e’	7.55 ± 1.81	8.68 ± 2.69	0.026	7.25 ± 1.84	8.16 ± 2.53	0.116
LAVI_max_ (mL/m^2^)	28.20 ± 6.72	30.00 ± 9.25	0.604	28.38 ± 6.25	29.27 ± 8.55	0.646
RV systolic function, volumes, and pressure						
TAPSE (mm)	22.40 ± 3.77	21.48 ± 4.65	0.425	22.47 ± 3.87	21.83 ± 3.77	0.523
RV S’ (m/s)	11.51 ± 1.60	11.93 ± 2.09	0.427	11.70 ± 1.67	12.10 ± 2.01	0.399
RVEDV (mL/m^2^)	36.67 ± 13.08	40.70 ± 20.04	0.784	39.50 ± 13.55	44.52 ± 20.02	0.263
RVESV (mL/m^2^)	17.29 ± 7.36	19.30 ± 10.08	0.446	18.67 ± 7.56	21.31 ± 10.49	0.270
RVEF (%)	52.98 ± 9.21	52.30 ± 10.15	0.753	53.07 ± 7.94	51.97 ± 10.96	0.659
RVEDA (cm^2^)	17.46 ± 3.92	18.61 ± 5.37	0.626	18.41 ± 3.93	19.80 ± 5.29	0.255
RVESA (cm^2^)	10.35 ± 2.69	11.15 ± 3.36	0.339	10.90 ± 2.73	11.87 ± 3.35	0.227
RV FAC (%)	40.81 ± 6.70	39.79 ± 7.98	0.533	41.02 ± 5.81	39.77 ± 8.33	0.505
RVSP (mmHg)	20.41 ± 5.18	22.63 ± 5.17	0.129	19.80 ± 3.35	21.55 ± 5.03	0.237
RV septal strain (%)	18.15 ± 3.47	17.18 ± 3.50	0.216	18.31 ± 3.28	17.88 ± 3.10	0.609
RV FWS (%)	27.32 ± 4.56	24.05 ± 5.89	0.008	26.86 ± 3.68	25.08 ± 5.40	0.151
RV global strain (%)	22.73 ± 3.27	20.62 ± 4.03	0.013	22.58 ± 2.85	21.46 ± 3.47	0.188
Abnormal RV FWS (<20%)	0	11 (31.4)	<0.001	0	6 (23.1)	0.005
Abnormal RV global strain (<17%)	0	9 (25.7)	<0.001	0	4 (15.4)	0.026

Mean ± standard deviation for continuous variables. Number (percentage) for categorical variables. Seven patients had sub-optimal RV focused images, and therefore were excluded from RV strain analysis; however, all other parameters were collected and included in table. FAC, fractional area change; FWS, free wall strain; GLS, global longitudinal strain; IVS, interventricular septum; LAVI_max_, maximum left atrial volume indexed; LV, left ventricular; LVEDD, left ventricular end diastolic diameter; LVEDV, left ventricular end diastolic volume; LVEF, left ventricular ejection fraction; LVESD, left ventricular end systolic diameter; LVESV, left ventricular end systolic volume; PW, posterior wall; RV, right ventricular; RVEDA, right ventricular end diastolic area; RVEDV, right ventricular end diastolic volume; RVEF, right ventricular ejection fraction; RVESA, right ventricular end systolic area; RVESV, right ventricular end systolic volume; RV S’, right ventricular systolic excursion velocity; RVSP, right ventricular systolic pressure; TAPSE, tricuspid annular plane systolic excursion.

**Table 3:** Demographic, clinical, and echocardiographic data of COVID-19 patient group stratified by LV GLS cut-off ≥16% versus <16%.

Parameters	LV GLS		
	≥16% (Normal) *n* = 36	<16% (Abnormal) *n* = 6	*p* Value
Age (y)	47.92 ± 17.50	39.83 ± 11.79	0.284
Female	25 (69.4)	2 (33.3)	0.087
BMI (kg/m^2^)	27.97 ± 5.55	28.41 ± 4.95	0.857
Diabetes	1 (2.8)	1 (16.7)	0.139
Hypertensive	6 (16.7)	0	0.280
Hypertensive and diabetic	4 (11.1)	0	0.391
Atrial fibrillation	2 (5.6)	0	0.554
Ischemic heart disease	1 (2.8)	0	0.679
Admitted to hospital	20 (55.6)	3 (50)	0.800
Admitted to ICU	3 (8.3)	3 (50)	0.007
Required oxygen	7 (19.4)	3 (50)	0.104
Invasive respiratory support	1 (2.8)	2 (33.3)	0.007
PW (mm)	8.67 ± 1.60	9.33 ± 1.21	0.281
IVS (mm)	8.92 ± 1.75	10.67 ± 2.16	0.034
LV mass (g/m^2^)	70.11 ± 17.76	94.08 ± 39.63	0.069
LVEDV (mL/m^2^)	44.16 ± 15.10	61.97 ± 22.93	0.017
LVESV (mL/m^2^)	17.34 ± 6.49	28.33 ± 16.63	0.053
LVEF (%)	60.81 ± 4.71	56.50 ± 8.19	0.208
TAPSE (mm)	21.48 ± 4.91	21.50 ± 2.88	0.847
RV S’ (m/s)	11.88 ± 1.97	12.17 ± 2.93	0.763
RVEDA (cm^2^)	17.94 ± 5.02	22.42 ± 6.19	0.065
RVESA (cm^2^)	10.59 ± 2.90	14.32 ± 4.25	0.027
FAC (%)	40.43 ± 8.19	36.17 ± 5.98	0.232
RV FWS (%)	24.44 ± 6.13	21.68 ± 3.68	0.338
Peak E (cm/s)	73.69 ± 19.12	69.83 ± 10.25	0.634
Peak A (cm/s)	68.81 ± 16.91	60.00 ± 16.31	0.297
Septal e’ (cm/s)	7.89 ± 2.94	8.50 ± 2.59	0.636
Lateral e’ (cm/s)	10.58 ± 4.31	11.33 ± 7.00	0.875
E/mean e’	8.69 ± 2.59	8.67 ± 3.50	0.987
LAVI_max_ (mL/m^2^)	29.17 ± 8.81	35.00 ± 11.10	0.155

Mean ± standard deviation for continuous variables; number (column percentage) for categorical variables. BMI, body mass index; FAC, fractional area change; FWS, free wall strain; GLS, global longitudinal strain; ICU, intensive care unit; IVS, interventricular septum; LAVI_max_, maximum left atrial volume indexed; LV, left ventricular; LVEDV, left ventricular end diastolic volume; LVEF, left ventricular ejection fraction; LVESV, left ventricular end systolic volume; PW, posterior wall; RV, right ventricular; RVEDA, right ventricular end diastolic area; RVESA, right ventricular end systolic area; RV S’, right ventricular systolic excursion velocity; TAPSE, tricuspid annular plane systolic excursion.

**Table 4:** Demographic, clinical, and echocardiographic data of COVID-19 patients stratified by RV FWS cut-off ≥20% versus <20%.

Parameters	RV FWS		
	≥20% (Normal) *n* = 24	<20% (Abnormal) *n* = 11	*p* Value
Age (y)	41.46 ± 15.85	53.09 ± 16.96	0.057
Female	15 (62.5)	7 (63.6)	1.000
BMI (kg/m^2^)	26.44 ± 4.65	29.31 ± 4.411	0.094
Diabetes	1 (4.2)	1 (9.1)	0.536
Hypertensive	2 (8.3)	3 (27.3)	0.297
Hypertensive and diabetic	1 (4.2)	1 (9.1)	0.536
Atrial fibrillation	0 (0)	1 (9.1)	0.314
Ischemic heart disease	1 (4.2)	0 (0)	1.000
Admitted to hospital	11 (45.8)	6 (54.5)	0.725
Admitted to ICU	2 (8.3)	2 (18.2)	0.575
Required oxygen	3 (12.5)	3 (27.3)	0.352
Invasive respiratory support	0 (0)	2 (18.2)	0.092
PW (mm)	8.54 ± 1.69	9.00 ± 1.55	0.451
IVS (mm)	8.75 ± 2.03	9.64 ± 1.29	0.194
LV mass (g/m^2^)	74.06 ± 27.81	71.26 ± 18.02	0.986
LVEDV (mL/m^2^)	50.95 ± 17.40	41.84 ± 16.95	0.072
LVESV (mL/m^2^)	21.25 ± 10.41	16.63 ± 6.78	0.142
LVEF (%)	59.50 ± 6.12	60.18 ± 3.92	0.738
LV GLS (%)	18.42 ± 2.62	18.27 ± 2.21	0.877
TAPSE (mm)	22.22 ± 5.73	19.82 ± 2.48	0.027
RV S’ (m/s)	12.46 ± 2.13	10.60 ± 1.84	0.025
RVEDA (cm^2^)	19.48 ± 5.67	17.34 ± 5.02	0.299
RVESA (cm^2^)	11.43 ± 3.62	10.56 ± 3.08	0.713
FAC (%)	41.18 ± 7.53	38.49 ± 9.98	0.383
Peak E (cm/s)	79.21 ± 17.78	61.36 ± 10.38	0.004
Peak A (cm/s)	63.63 ± 16.96	70.27 ± 16.61	0.287
Septal e’ (cm/s)	8.88 ± 2.97	7.00 ± 2.53	0.078
Lateral e’ (cm/s)	11.83 ± 4.79	9.27 ± 4.73	0.107
E/mean e’	8.41 ± 2.54	8.27 ± 2.10	0.820
LAVI_max_ (mL/m^2^)	31.96 ± 9.65	28.45 ± 9.92	0.224

Mean ± standard deviation for continuous variables; number (column percentage) for categorical variables. BMI, body mass index; FAC, fractional area change; FWS, free wall strain; GLS, global longitudinal strain; ICU, intensive care unit; IVS, interventricular septum; LAVI_max_, maximum left atrial volume indexed; LV, left ventricular; LVEDV, left ventricular end diastolic volume; LVEF, left ventricular ejection fraction; LVESV, left ventricular end systolic volume; PW, posterior wall; RV, right ventricular; RVEDA, right ventricular end diastolic area; RVESA, right ventricular end systolic area; RV S’, right ventricular systolic excursion velocity; TAPSE, tricuspid annular plane systolic excursion.

**Table 5:** Odds ratio of LV and RV strain parameters in predicting routinely used echocardiographic functional parameters in the COVID-19 cohort.

	OR	Confidence Interval
OR for abnormal LV GLS to predict LVEF < 55%	8.50	0.93–78.02
OR for abnormal RV FWS to predict RV S’ < 9.5 m/s	9.86	0.88–110.43
OR for abnormal global RV strain to predict RV S’ < 9.5 m/s	4.00	0.46–34.49

FWS, free wall strain; GLS, global longitudinal strain; LV, left ventricular; LVEF, left ventricular ejection fraction; OR, odds ratio; RV, right ventricular; RV S’, right ventricular systolic excursion velocity.

## Data Availability

The data presented in this study are available on request from the corresponding author. The data are not publicly available due to privacy reasons.

## Supplementary Materials

The following supporting information can be downloaded at: https://www.mdpi.com/article/10.3390/jcdd10080349/s1, Table S1: Univariate and multivariate analysis of clinical and echocardiographic parameters against LV GLS in the entire group (*n* = 84); Table S2: Univariate and multivariate analysis of clinical and echocardiographic parameters against RV FWS in the entire group (*n* = 84); Table S3: Drugs administered to COVID-19 patients stratified by LV GLS and RV FWS. Please refer to non-published material file.
