# Supplementary material for: Echocardiographic Assessment in Patients Recovered from Acute COVID-19 Illness
Source: J Cardiovasc Dev Dis. 2023 Aug 15;10(8):349. doi: 10.3390/jcdd10080349 (PMC10456092; doi:10.3390/jcdd10080349)
Supplement: Supplementary file 1 [file jcdd-10-00349-s001.zip › jcdd-2520149-supplementary.pdf]

Supplementary tables

Table S1. Univariate and multivariate analysis of clinical and echocardiographic parameters against LV GLS in the entire group (n=84).

Parameters	Univariate		Multivariate	
	R (CI)	p value	Standardized β	p value
COVID-19 infection	-0.333 (-0.511 to -0.128)	0.002	-0.265	0.012
Sex	-0.310 (-0.492 to -0.102)	0.004	-0.095	0.434
Admitted to ICU	-0.275 (-0.462 to -0.065)	0.011	-0.180	0.075
BMI	-0.095 (-0.303 to -0.122)	0.390	0.060	0.594
LV mass	-0.261 (-0.450 to -0.049)	0.017	0.015	0.906
LVEDV	-0.312 (-0.494 to -0.105)	0.004	-0.194	0.089
RVEDA	-0.412 (-0.577 to -0.214)	<0.001	-0.269	0.056

Variables selected based on univariate analysis with GLS. BMI, body mass index; CI, confidence interval; ICU, intensive care unit; LV, left ventricular; LVEDV, left ventricular end diastolic volume; RVEDA, right ventricular end diastolic area.

Table S2. Univariate and multivariate analysis of clinical and echocardiographic parameters against RV FWS in the entire group (n=84).

Parameters	Univariate		Multivariate	
	R (CI)	p value	Standardized β	p value
COVID-19 infection	-0.302 (-0.493 to -0.084)	0.008	-0.226	0.034
Invasive respiratory support	-0.264 (-0.461 to -0.042)	0.020	-0.182	0.084
DBP	-0.350 (-0.532 to -0.137)	0.002	-0.277	0.010
FAC	0.217 (0.050 to 0.467)	0.017	0.237	0.022
BMI	0.226 (-0.370 to 0.067)	0.192	-0.015	0.891

Variables selected based on univariate analysis with RV FWS. BMI, body mass index; CI, confidence interval; DBP, diastolic blood pressure; FAC, fractional area change.

Table S3. Drugs administered to COVID-19 patients stratified by LV GLS and RV FWS.

	LV GLS		RV FWS	
	≥16% (normal)	<16% (abnormal)	≥20% (normal)	<20% (abnormal)
	n=36	n=6	n=24	n=11
Medication	6 (16.7%)	3 (50%)	3 (12.5%)	3 (27.3%)
Broad-Spectrum A/B	5 (13.9%)	2 (33.3%)	1 (4.2%)	3 (27.3%)
Hydroxychloroquine	0	2 (33.3%)	0	1 (9.1%)
Antivirals	1 (2.8%)	1 (16.7%)	0	2 (18.2%)
Remdesivir	2 (5.6%)	1 (16.7%)	2 (8.3%)	0
Dexamethasone	3 (8.3%)	1 (16.7%)	3 (12.5%)	0
